# Molecular Insights into the Fungus-Specific Serine/Threonine Protein Phosphatase Z1 in *Candida albicans*

**DOI:** 10.1128/mBio.00872-16

**Published:** 2016-08-30

**Authors:** Emily Chen, Meng S. Choy, Katalin Petrényi, Zoltán Kónya, Ferenc Erdődi, Viktor Dombrádi, Wolfgang Peti, Rebecca Page

**Affiliations:** a Department of Molecular Biology, Cell Biology and Biochemistry, Brown University, Providence, Rhode Island, USA; b Department of Molecular Pharmacology, Physiology and Biotechnology, Brown University, Providence, Rhode Island, USA; c Department of Medical Chemistry, Faculty of Medicine, University of Debrecen, Debrecen, Hungary; d Department of Chemistry, Brown University, Providence, Rhode Island, USA

## Abstract

The opportunistic pathogen *Candida* is one of the most common causes of nosocomial bloodstream infections. Because candidemia is associated with high mortality rates and because the incidences of multidrug-resistant *Candida* are increasing, efforts to identify novel targets for the development of potent antifungals are warranted. Here, we describe the structure and function of the first member of a family of protein phosphatases that is specific to fungi, protein phosphatase Z1 (PPZ1) from *Candida albicans*. We show that PPZ1 not only is active but also is as susceptible to inhibition by the cyclic peptide inhibitor microcystin-LR as its most similar human homolog, protein phosphatase 1α (PP1α [GLC7 in the yeast *Saccharomyces cerevisiae*]). Unexpectedly, we also discovered that, despite its 66% sequence identity to PP1α, the catalytic domain of PPZ1 contains novel structural elements that are not present in PP1α. We then used activity and pulldown assays to show that these structural differences block a large subset of PP1/GLC7 regulatory proteins from effectively binding PPZ1, demonstrating that PPZ1 does not compete with GLC7 for its regulatory proteins. Equally important, these unique structural elements provide new pockets suitable for the development of PPZ1-specific inhibitors. Together, these studies not only reveal why PPZ1 does not negatively impact GLC7 activity *in vivo* but also demonstrate that the family of fungus-specific phosphatases—especially PPZ1 from *C. albicans*—are highly suitable targets for the development of novel drugs that specifically target *C. albicans* without cross-reacting with human phosphatases.

## IMPORTANCE

*Candida albicans* is a medically important human pathogen that is the most common cause of fungal infections in humans. In particular, approximately 46,000 cases of health care-associated candidiasis occur each year in the United States. Because these infections are associated with high mortality rates and because multiple species of *Candida* are becoming increasingly resistant to antifungals, there are increasing efforts to identify novel targets that are essential for *C. albicans* virulence. Here we use structural and biochemical approaches to elucidate how a member of a fungus-specific family of enzymes, serine/threonine phosphatase PPZ1, functions in *C. albicans*. We discovered multiple unique features of PPZ1 that explain why it does not cross-react with, and in turn compete for, PP1-specific regulators, a long-standing question in the field. Most importantly, however, these unique features identified PPZ1 as a potential target for the development of novel antifungal therapeutics that will provide new, safe, and potent treatments for candidiasis in humans.

## INTRODUCTION

*Candida albicans* is an opportunistic fungal pathogen that causes candidemia and is the most common cause of health care-associated *Candida* bloodstream infections in the United States (1). In the past, the majority of candidemia patients were immunocompromised (i.e., individuals with HIV or transplant recipients on immunosuppressant drugs, among others). However, the numbers of nonimmunocompromised patients contracting candidemia have been steadily increasing, with an estimate of 7,000 to 28,000 patients contracting nosocomial candidemia annually (2). Because the mortality rate of candidemia is 40%, these infections result in ~2,800 to 11,200 deaths per year. Unfortunately, these numbers are expected to increase as multiple species of *Candida* are becoming increasingly resistant to antifungal medications, including fluconazole and echinocandins (3). Although a combination of the well-established calcineurin (CN) drugs FK-506 and cyclosporine (CSA) given with the fungal inhibitor fluconazole has resulted in the very potent killing of *C. albicans* (4), the immunosuppressant functions of FK-506 and CSA make their use in humans problematic. Furthermore, *C. albicans*-specific CN inhibitors are unlikely to be achievable due to the 100% conservation of the CN active and substrate binding sites (5). Given the pressing need for new, potent antifungals, efforts to identify novel protein targets that are essential for virulence and unique to *C. albicans* are warranted.

**FIG 1:**
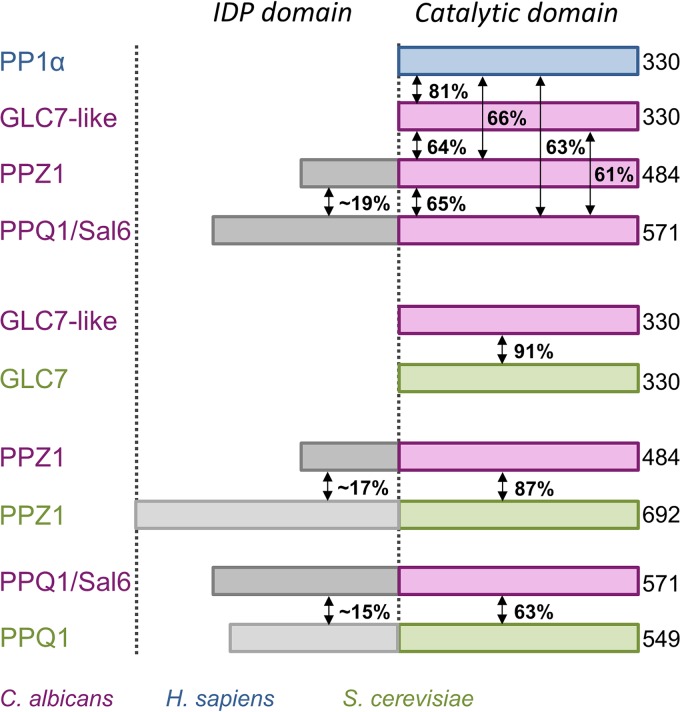
*Ca*PPZ1 is a PP1-like phosphatase. Domain architecture of the PP1-like phosphatases in *C. albicans* (GLC7-like, PPZ1, and PPQ1/Sal6 [pink]), *S. cerevisiae* (green), and *Homo sapiens* PP1α (blue). Fungus-specific PP1-like phosphatases have an N-terminal intrinsically disordered protein domain (IDP [gray]) in addition to a structured C-terminal catalytic domain (pink/green).

Eukaryotes contain multiple genes that encode serine/threonine protein phosphatase 1 (PP1 [in humans, PP1α, PP1β, and PP1γ]). PP1 regulates diverse and essential biological processes by dephosphorylating a variety of protein substrates. Although the intrinsic substrate specificity of PP1 is very low, by interacting with regulatory proteins to form distinct holoenzymes (~200 biochemically confirmed PP1 interactors), PP1 achieves high specificity (6–8). More than two decades ago, it was discovered that budding yeast (*Saccharomyces cerevisiae*), fission yeast (*Schizosaccharomyces pombe*), and the opportunistic fungus *Candida albicans* also carry a PP1 gene, coding for GLC7, Dis2, and GLC7-like, respectively (Fig. 1). There is ~80% sequence identity between *C. albicans* GLC7 and human PP1 isoforms (“GLC7” will be used here to refer to the PP1 homolog in fungal species) (9). Like PP1, GLC7 controls a plethora of essential biological processes, and its activity is regulated by its interaction with multiple regulatory proteins, many of which are conserved in humans (8). However, it was also discovered that fungi express a unique family of PP1-like genes, which include those coding for PPZ1 and PPQ1/Sal6 (10, 11). Unlike GLC7, these families of fungus-specific PP1-like phosphatases consist of two distinct domains: an N-terminal domain that is enriched in serines and is predicted to be unstructured (i.e., a member of the intrinsically disordered protein [IDP] family) and a C-terminal catalytic domain that has high sequence similarity to GLC7 (Fig. 1). This suggests that the fungus-specific phosphatases may also bind the GLC7-specific regulatory proteins. This was confirmed in *S. cerevisiae*, in which yeast two-hybrid studies demonstrated that *S. cerevisiae* PPZ1 (*Sc*PPZ1) binds some, but not all, *S. cerevisiae* GLC7 (*Sc*GLC7)-specific regulatory proteins (12). However, the molecular determinants that explain why fungus-specific phosphatases bind only a subset of the GLC7-specific regulators are still largely unknown.

Furthermore, not only is PPZ1 important for cation homeostasis and cell wall biosynthesis, it is also critical for *C. albicans* virulence (13, 14). Namely, deletion of the Ppz1 gene reduces the ability of *C. albicans* to infect mice, a function that can be rescued by the reintegration of a single copy of the Ppz1 gene. In addition, PPZ1 has also been shown to play a role in the morphological changes in *C. albicans* associated with infectivity: that is, the transition from the yeast to the hyphal form. This is because the deletion of Ppz1 reduces the rate of hyphal growth (15). Together, these data suggest that the specific inhibition of PPZ1 will prevent this morphological transition and, as a consequence, block *C. albicans* infectivity without killing the commensal pathogen. The fungistatic effect of such a treatment would be more beneficial than eliminating *C. albicans* altogether as the latter might result in uncontrolled bacterial proliferation. Here, we used X-ray crystallography and biochemistry to elucidate the structural and functional characteristics of PPZ1 that are unique to the fungus-specific family of phosphatases. We discovered novel structural elements in PPZ1 that not only explain why PPZ1 binds less effectively to GLC7 regulators but also define new interaction surfaces that may be leveraged for the development of novel, effective antifungal therapeutics.

## RESULTS

### PPZ1_cat_ is an active phosphatase with an atypical C terminus.

Like other fungus-specific phosphatases, *C. albicans* PPZ1 (484 amino acids [aa], 54.4 kDa) has two domains: an N-terminal intrinsically disordered protein (IDP) domain (aa 1 to 170 [PPZ1_Nterm_]) and a C-terminal catalytic domain (aa 171 to 484 [PPZ1_cat_]) (14, 16). PPZ1_cat_ and human PP1α exhibit 66% sequence identity throughout their catalytic domains, and the 6 residues that coordinate the active site metals are 100% conserved (Fig. 1; see Fig. S1 in the supplemental material). Accordingly, full-length PPZ1 (PPZ1_FL_) and its individual domains (PPZ1_Nterm_ and PPZ1_cat_) are readily isolated from *Escherichia coli*. In addition, PPZ1_FL_ and PPZ1_cat_ are active, as they effectively dephosphorylate a small molecule substrate mimetic (*p*-nitrophenyl phosphate [pNPP]) (see Fig. S2 in the supplemental material). Furthermore, their activities are identical to that of human PP1α purified from *E. coli* (8) or PP1c (a mixture of PP1 isoforms α, β, and γ) purified from rabbit muscle (17). However, unlike PP1α, which expresses solubly and crystallizes readily without its C-terminal disordered residues (residues 301 to 330) (8), a PPZ1 construct truncated at the corresponding residue (PPZ1_cat_Δ466–484) is largely insoluble compared to PPZ1_cat_ (>10-fold reduction in yield). This suggests that the PPZ1_cat_ C-terminal region is critical for folding and/or stability. Consistent with this conclusion, the secondary structure prediction program PSIPRED (18) predicts that these residues are not disordered as they are in PP1α but instead form an α-helix (see Fig. S3 in the supplemental material).

### The C-terminal residues of PPZ1_cat_ are structured and form an α-helix.

**FIG 2:**
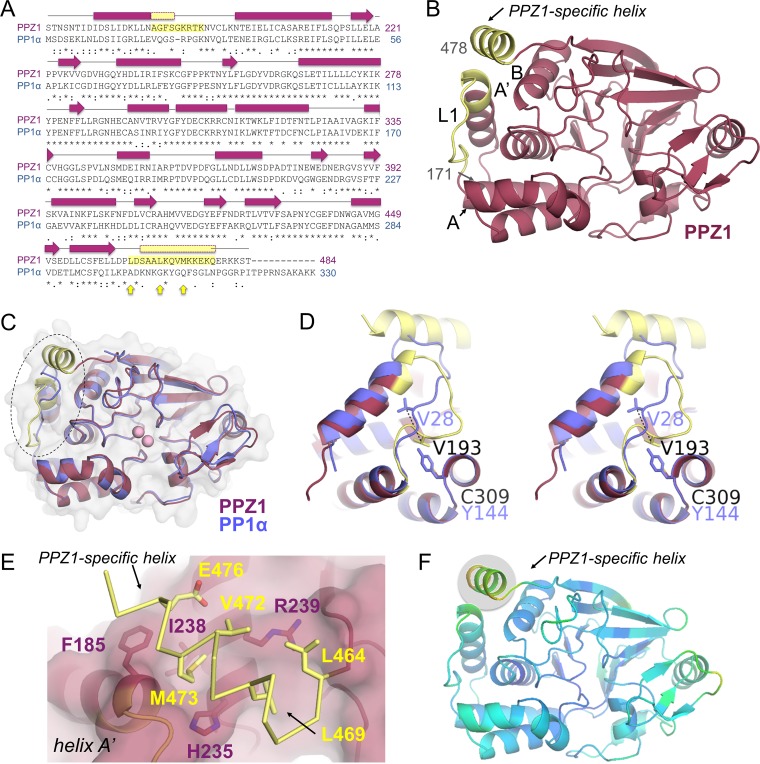
The PPZ1-specific C-terminal helix. (A) Sequence alignment of *Ca*PPZ1 (pink) and *Homo sapiens* PP1 (*Hs*PP1α [blue]) with the observed secondary structural elements indicated above the sequence. Identical residues are indicated by a star, similar residues are indicated by a colon, less similar residues are indicated by a period, and dissimilar residues are indicated by a blank space. Resides from loop 1 (L1) and the PPZ1-specific helix are highlighted in yellow. Arrows (yellow) indicate the hydrophobic residues in the PPZ1-specific helix that are not present in PP1α. (B) The structure of PPZ1 is shown with the secondary structural elements discussed in the text labeled. (C) Overlay of PPZ1 (pink and yellow, as in panel B) and PP1α (blue). The change in conformation of loop L1 between the two structures is indicated by a dashed circle. (D) Stereo image of the overlay between L1 from PPZ1 and PP1α, colored as in panel C. (E) Interactions between the PPZ1-specific C-terminal helix (yellow) and the widened PPZ1-specific helix binding pocket (coral). Residues that make key interactions are shown as sticks and labeled. (F) PPZ1 colored according to residue B-factors, with yellow and green shading indicating higher B-factors.

In order to understand the molecular consequences of the sequence differences between PPZ1 and PP1α and the role of the PPZ1 C-terminal residues in PPZ1 function, we determined the 3-dimensional crystal structure of PPZ1_cat_ to 2.61 Å resolution (Table 1). The PPZ1_cat_ structure includes residues 171 to 478. (The electron density for the last 6 residues, 479 to 484, was not observed, and thus they were not modeled.) As expected, the PPZ1_cat_ structure adopts the canonical PP1 fold (Fig. 2A and B), comprising a mixed α/β protein, whose central loops are positioned to coordinate the active site metals. However, the structures are not identical. The root mean square deviation (RMSD) between PP1α (PDB no. 4MOV [19]) and PPZ1_cat_ is 1.23 Å (backbone). The most significant difference is in the N-terminal region, where the PPZ1_cat_ helix A′ extends for one more turn compared with PP1α and the loop connecting helix A′ to helix A (L1) adopts a conformation distinct from that in PP1α (Fig. 2B to D). (The RMSD of L1 between PPZ1_cat_ and PP1α is 2.99 Å.) Although the sequences of L1 between PPZ1_cat_ and PP1α are only 33% identical, this change of conformation is primarily due to a single amino acid change in helix C, Tyr144_PP1α_ to Cys309_PPZ1_. The much smaller cysteine side chain creates a hydrophobic pocket into which the side chain of Val193_PPZ1_ binds (Fig. 2D). Because this pocket is not present in PP1α, the corresponding PP1α residue, Val28_PP1α_, interacts directly with helix A′. As a consequence, the Cβ atoms of the two side chains are separated by 5.6 Å between the two structures. The rest of the PPZ1_cat_ L1 conformation changes to accommodate this new interaction, which in turn widens the pocket defined by helix A′, L1, and helix B (referred to here as the “Z1-helix binding pocket”).

**TABLE 1:** Data collection and refinement statistics

Parameter	Value(s) for^a^:	
	*Ca*PPZ1	*Ca*PPZ1–microcystin-LR
Data collection		
Space group	C222_1_	P3_2_21
Cell dimensions		
*a*, *b*, *c* (Å)	145.0, 183.7, 69.0	50.7, 50.7, 201.1
α, β, γ (°)	90, 90, 90	90, 90, 120
Resolution (Å)	50.0–2.61 (2.66–2.61)	50.0–2.40 (2.44–2.40)
*R*_merge_ (%)	13.3 (84.5)	6.6 (19.7)
*I*/σ〈*I*〉	14.1 (2.1)	30.6 (4.6)
Completeness (%)	99.9 (100.0)	98.5 (84.2)
Redundancy	5.6 (5.6)	6.1 (2.7)

Refinement		
Resolution (Å)	36.24–2.61	43.9–2.40
No. of reflections	28,311	12,283
*R*_work_/*R*_free_	19.4/22.2	17.9/23.5
No. of atoms		
Protein	4,922	2,314
Ligand/ion	32	23
Microcystin-LR	NA^b^	71
Water	220	50
B-factors		
Protein	33.0	30.8
Ligand/ion	29.9	46.6
Microcystin	NA	35.5
Water	30.9	30.1
RMSDs		
Bond lengths (Å)	0.002	0.003
Bond angles (°)	0.72	0.57
Ramachandran plot (%)		
Favored regions	96.1	96.5
Allowed regions	3.8	3.5
Disallowed regions	0.2	0.0
PDB accession no.	5JPE	5JPF

a Values in parentheses are for the highest-resolution shell.

b NA, not applicable.

The newly widened Z1-helix binding pocket results in the second significant structural difference between PPZ1_cat_ and PP1α: the C-terminal residues of PPZ1_cat_ are not disordered like they are in PP1α, but instead form an α-helix that nestles into this newly widened Z1-helix binding pocket (Fig. 2B to E). This interaction is stabilized by hydrophobic interactions between the C-terminal helix and residues from helices A′ and B, with the interaction centered on PPZ1 C-terminal helix residue Met473_PPZ1_ (Fig. 2E). This residue is completely buried from solvent via interactions with Leu464_PPZ1_, Leu469_PPZ1_, and Val472_PPZ1_ from the C-terminal helix as well as by interactions with Phe185_PPZ1_ from helix A′ and His235_PPZ1_, Ile238_PPZ1_, and Arg239_PPZ1_ from helix B. The majority of these residues are not conserved in PP1α. In particular, only a single residue (underlined) between the PPZ1 C-terminal helix and the C-terminal disordered tail of PP1α is conserved (PPZ1, ^466^SAALKQVMKKEKQ^478^; PP1α, ^301^KNKGKYGQFSGLN^313^), with the sequence of PPZ1 consisting of multiple hydrophobic residues and no helix-disrupting glycines (Leu469_PPZ1_, Val472_PPZ1_, and Met473_PPZ1_ versus Gly304_PP1α_, Gly307_PP1α_, and Gly311_PP1α_, respectively), rationalizing why the corresponding residues in PP1α are unstructured. Finally, the experimental B-factors for residues in the Z1-helix are higher than the rest of PPZ1_cat_ (Fig. 2F), suggesting that the Z1-helix is more dynamic than the rest of the PPZ1_cat_.

### The Z1-helix is dynamic.

**FIG 3:**
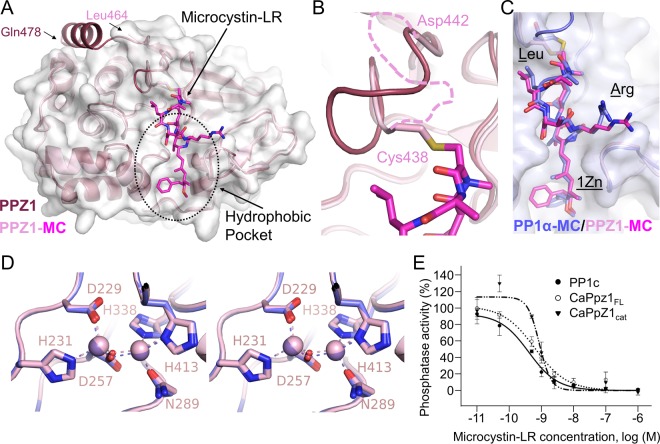
The PPZ1-specific helix is dynamic. (A) Overlay of free PPZ1 (dark pink) and MC-bound PPZ1 (light pink). The C-terminal residues of both proteins are labeled. The gray surface corresponds to the PPZ1-MC complex, illustrating that the PPZ1-specific helix was not ordered and thus not modeled (see the text for details). The PPZ1 hydrophobic pocket is indicated by a dashed circle. (B) Enlarged view of the covalent bond between Cys438 and MC, resulting in a change in conformation of the β12-β13 loop. (Residues 439 to 441 were not visible in the PPZ1-MC structure and are indicated by a pink dashed line.) (C) Overlay of MC from PPZ1 (light pink) and PP1α (blue). The Leu and Arg residues in microcystin-LR are labeled, as is the 1Zn [(2S,3S,4E,6E,8S,9S)-3-amino-9-methoxy-2,6,8-trimethyl-10-phenyldeca-4,6-dienoic acid]. (D) Stereo image of the PPZ1 and PP1α metal-bound active sites. Bound Mn^2+^ ions are shown as spheres. (E) Dose-response curves reporting the inhibition of PP1c, PPZ1_FL_, and PPZ1_cat_ activities by the MC toxin; ^32^P-labeled MLC20 was used as a substrate. The phosphatase activities of each enzyme without toxin were set to 100% activity. The data represent the means ± standard errors (SE) from 3 experiments.

We also determined the structure of PPZ1_cat_ bound to the marine toxin microcystin-LR (MC [PPZ1_cat_-MC]) (Table 1), a potent cyclic peptide inhibitor of PP1 (20). Both the MC-free and MC-bound structures of PPZ1_cat_ are highly similar, with an RMSD of 0.54 Å (Fig. 3A). The largest difference is in the β12-β13 loop, which contains Cys438_PPZ1_ (Fig. 3B). This cysteine forms a covalent bond with the bound MC identical to that observed in the PP1α-MC complex (21). This causes the ^438^CGEFD^442^ loop to change conformation and become more dynamic (residues ^439^GEF^441^ were not modeled in the PPZ1_cat_-MC structure due to a lack of density). Unexpectedly, the Z1-helix is also no longer ordered in the PPZ1_cat_-MC structure (Fig. 3A). This was not due to MC binding, but instead the Z1-helix was displaced by a symmetry-related molecule in the crystal. The observation that the Z1-helix can be displaced supports the observation that the Z1-helix is more dynamic than the rest of the PPZ1_cat_. Importantly, the positions of L1 are identical between the PPZ1_cat_ and PPZ1_cat_-MC structures, demonstrating that the conformation of L1 is intrinsic to PPZ1_cat_ itself and not a consequence of Z1-helix binding.

MC binds the active site of PPZ1_cat_ essentially identically to that observed in PP1α (Fig. 3C). Furthermore, the active sites are also identical (Fig. 3D). The bulk of the MC binds the PPZ1_cat_ hydrophobic binding pocket (Fig. 3A), while the remainder covers and, as a consequence, blocks the active site. The largest difference between the conformations of MC in PP1α-MC and PPZ1_cat_-MC is a change of the rotomer conformation of the 1Zn residue phenyl group and the Arg guanidinium group (Fig. 3C). The orientation observed in PPZ1_cat_-MC is favored because a malonate ion from the crystallization mother liquor is bound in the rotomer position populated in the PP1α structure; thus both positions are expected to be equally likely in the absence of malonate. Furthermore, because the residues that mediate MC binding are highly conserved between PPZ1_cat_ and PP1α (79% identical and 90% similar [see Fig. S1 in the supplemental material]), MC is predicted to inhibit their activities with similar 50% inhibitory concentration (IC_50_) values. We tested this *in vitro* using both small molecule (pNPP)- and peptide (myosin light chain [pMLC])-based substrates, which show that the IC_50_ values of MC for all constructs are essentially identical (IC_50_s for pMLC of 0.96 nM for PPZ1_FL_, 0.72 nM for PPZ1_cat_, and 0.43 nM for PP1c, and IC_50_s for pNPP of 11.8 nM for PPZ1_cat_ and 4.4 nM for PP1α) (Fig. 3E; see Fig. S2 in the supplemental material).

### The sequence and structural conservation of regulatory protein binding pockets in PPZ1_cat_ is highly variable.

**FIG 4:**
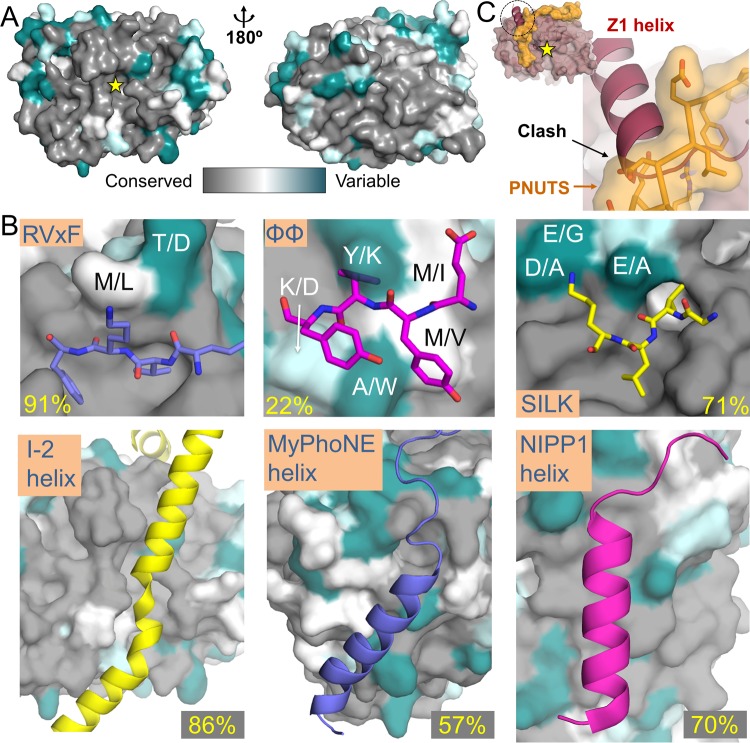
PP1 regulatory protein binding pockets are differentially conserved in PPZ1. (A) Sequence conservation between PPZ1 and PP1α mapped onto the surface of PPZ1. The yellow star indicates the location of the PP1 active site. (B) Close-up views of the RVxF, ΦΦ, SILK, I-2 helix, MyPhoNE helix, and NIPP1 helix binding pockets on PPZ1, with regulators that bind these pockets shown as sticks (RVxF, ΦΦ, and SILK) or a cartoon (I-2 helix, MyPhoNE helix, and NIPP1 helix). The sequence changes between PP1α/PPZ1 are indicated as in panel A. The sequence identity of the binding pockets is reported (see Fig. S1 in the supplemental material). (C) Overlay of a PP1 regulator (PNUTS [shown here in orange]) that bridges the ΦΦ and Arg motif binding pockets onto PPZ1_cat_ (rose). The clash between the regulator and Z1-specific helix is shown.

While PP1 exhibits broad specificity, it acts in a highly specific manner by forming stable complexes (holoenzymes) with a host of regulatory proteins that direct PP1 activity toward specific substrates and localize PP1 to specific regions of the cell (8, 22). Recent structural studies have revealed that PP1 binds these regulators using small linear interaction motifs (SLiMs) (7, 19, 23, 24). The most well-known is the RVxF SLiM, which is found in ~70% of all known regulators (22). Others include the MyPhoNE, SILK, ΦΦ, and Arg SLiMs (6, 7, 19, 23, 25). Mapping of the sequence differences onto the structure of PPZ1_cat_ shows that the bulk of the changes are distally located from the active site (Fig. 4A). This comparison also reveals that the RVxF interaction residues are 91% conserved between PPZ1 and PP1α (the only difference being a conservative Met290_PP1α_-to-Leu455_PPZ1_ substitution [Fig. 4B; see Fig. S1 in the supplemental material]), suggesting that all PP1 regulators that contain an RVxF sequence should bind PPZ1. Other sites are similarly conserved. For example, inhibitor-2 (I-2) is a specific protein inhibitor of PP1. In addition to an RVxF and SILK motif, it binds PP1 using a long helix, which binds across the PP1 active site (25). Like the RVxF motif, the I-2 helix binding pocket is highly conserved in PPZ1 (86% identical, 93% conserved Fig. 4B; see Fig. S1) suggesting that I-2 can bind productively to PPZ1. Likewise, the conservation of the NIPP1-helix interaction pocket (70% identical and 80% conserved [Fig. 4B; see Fig. S1]), also suggesting that this PP1 regulatory protein can bind productively to PPZ1_cat_.

In contrast, other SLiM binding pockets are much less conserved in PPZ1. For example, the SILK binding sites are only 71% identical and 79% similar between PPZ1 and PP1α (Fig. 4B; see Fig. S1 in the supplemental material). Three of the 4 amino acid differences result in a change from an acidic to uncharged residue (Glu56_PP1α_/Ala221_PPZ1_, Asp166_PP1α_/Ala331_PPZ1_, and Glu167_PP1α_/Gly332_PPZ1_). As these acidic residues coordinate the basic “K” of the SILK motif, the “K” may not be necessary for PPZ1 to bind SILK motif-containing proteins. The same is true for the MyPhoNE binding pocket (23) (Fig. 4B; see Fig. S1). While the majority of residues that define the pocket are largely similar—57% identical and 75% similar—multiple residues differ between the two proteins (Asp179_PP1α_/Val344_PPZ1_, Gln198_PP1α_/Phe363_PPZ1_, Gly215_PP1α_/Glu380_PPZ1_, and His237_PP1α_/Ser402_PPZ1_). The Gly215_PP1α_/Glu380_PPZ1_ substitution results in the largest clash in the superimposed structures, with the Glu380_PPZ1_ side chain clashing with that of Trp17_MYPT1_, suggesting that these changes impact MYPT1 binding.

The SLiM binding pocket that is most different between PP1α and PPZ1 is the ΦΦ interaction site, with an identity of 22% and a similarity of 67% (7, 19) (Fig. 4B; see Fig. S1 in the supplemental material). The most significant differences are the replacement of Tyr78_PP1α_, which defines the ΦΦ interaction pocket, with Lys243_PPZ1_ and the replacement of Ala279_PP1α_ with Trp444_PPZ1_. Both Lys243_PPZ1_ and Trp444_PPZ1_ are large, bulky residues that hinder access to the pocket. This suggests that PP1 regulators that contain a ΦΦ motif will bind PPZ1 with lower affinity than PP1α. Finally, although the Arg interaction pocket is perfectly conserved between both PP1α and PPZ1 (see Fig. S1), the presence of the Z1-helix in PPZ1 is expected to negatively impact the binding of regulators that use this site for binding, especially those that also use the ΦΦ binding pocket. Because the Z1-helix binds between these two sites, its presence will block access to the Arg site (Fig. 4C), reducing the ability of PPZ1 to bind regulators that contain these sequential SLiMs.

### PPZ1 binds only a subset of PP1 regulatory proteins.

**FIG 5:**
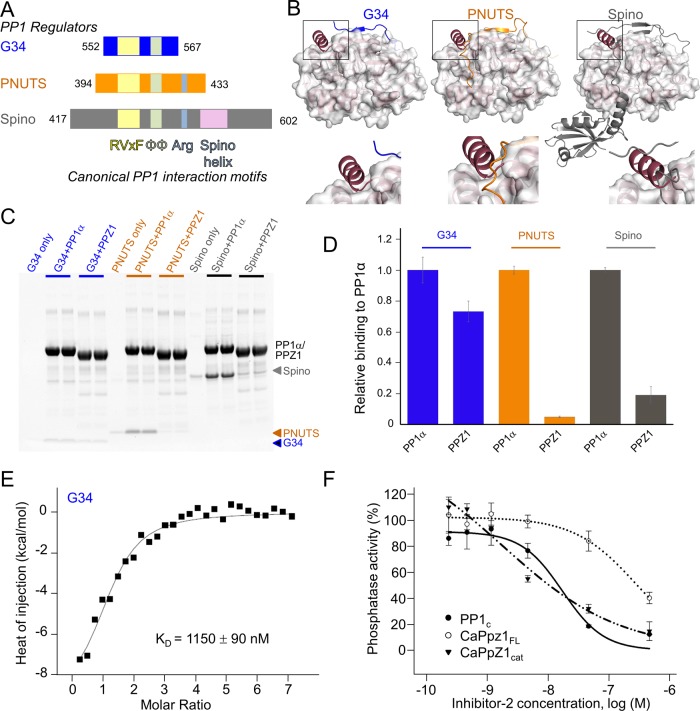
Most PP1 regulatory proteins bind to PPZ1 less effectively. (A) Domain structure of the regulators tested (G34, GADD34_552–567_; PNUTS, PNUTS_394–433_; Spino, spinophilin_417–602_) with their canonical PP1 interaction motifs highlighted in different colors. (B) Structures of the GADD34 (blue; PDB no. 4XPN [26]), PNUTS (orange; PDB no. 4MOY [19]), and spinophilin (gray; PDB no. 3EGG [27]) PP1 holoenzymes, with close-ups of the Z1 helix interaction pocket. (C) The relative binding of PP1α and PPZ1 with the regulators described in panel A was determined using a pulldown assay. (D) Densitometry analysis of the gel in panel B. (E) Binding isotherm of GADD34_552–567_ with PPZ1_cat_ (*K_D_*, 1,150 ± 90 nM). (F) Dose-response curves of phosphatase activity with increasing concentrations of I-2 with PP1_c_, PPZ1_cat_, and PPZ1_FL_ phosphatases. The data represent the means ± SE from 3 experiments.

To test if PPZ1 binds differentially to known PP1 regulators, we used pulldown assays with three well-characterized regulators—GADD34, PNUTS, and spinophilin (Fig. 5A and B). The minimal PP1-binding domains of these regulators bind PP1α with strong affinities (Equilibrium dissociation constant [*K_D_*] values are 62 nM for GADD34_552–567_ [26], 9.3 nM for PNUTS_394–433_ [19], and 8.7 nM for spinophilin_417–602_ [27]). As predicted, the pulldown assays show that PPZ1_cat_ binds GADD34 less effectively than PP1α (Fig. 5C and D). Because the RVxF site is nearly perfectly conserved between PP1 and PPZ1_cat_, this suggests that the weakened affinity is largely due to an inability of GADD34_552–567_ to productively bind the ΦΦ motif binding pocket. To quantify the reduction in binding, we used isothermal titration calorimetry (ITC). The affinity of GADD34 for PPZ1_cat_ decreases more than 19-fold, resulting in a *K_D_* of only 1,150 ± 90 nM (Fig. 5E). Consistent with this, peptides that contain only RVxF motifs bind PP1 with *K_D_*s in the micromolar range (28), similar to those observed for GADD34_552–567_ and PPZ1_cat_.

While the PP1 binding domain of GADD34 contains only an RVxF motif and ΦΦ motif, other regulators, such as PNUTS_394–433_ and spinophilin_417–602_ contain additional motifs that facilitate PP1 binding (PNUTS, RVxF-ΦΦ-Arg; spinophilin, RVxF–ΦΦ–Arg–spinophilin_417–602_–helix [Fig. 5A and B]). We used pulldown assays to determine if the altered ΦΦ binding pocket and the presence of the Z1-helix negatively impact the binding of these regulators to PPZ1 (Fig. 5C). The results show that PPZ1, compared to PP1α, does not effectively pull down either PNUTS or spinophilin (~85% less binding [Fig. 5C and D]). Furthermore, mutation of either the ΦΦ or Arg motif in spinophilin results in a similar reduction in PP1 binding, to levels similar to those observed for PPZ1 and wild-type spinophilin (see Fig. S4 in the supplemental material). Together, these data demonstrate that the altered ΦΦ-binding pocket coupled with the presence of the Z1-helix (Fig. 5B) negatively impacts the ability of PPZ1 to interact with regulators that require these sites for binding.

### The presence of the N-terminal IDP domain reduces the ability of I-2 to inhibit PPZ1.

Unlike GADD34, PNUTS, and spinophilin, I-2 does not bind PP1 at the ΦΦ- or PPZ1-specific binding pockets. Instead, it binds PP1 using the SILK–RVxF–I-2–helix interaction pockets (25). With the exception of the SILK motif binding pocket, these interaction sites in PPZ1 are largely conserved with those in PP1α. Furthermore, the change in the SILK binding pocket suggests that PPZ1 no longer has a strict requirement for the “K” residue (Fig. 4B). This suggests that I-2 will inhibit both PPZ1 and PP1α with equal potencies. To test this, we measured the ability of I-2 to bind and inhibit PPZ1 activity and compared it to that determined for PP1c. The data show that both PPZ1_cat_ and PP1c are inhibited by I-2 with nearly equivalent IC_50_s, measured to be 7 and 12 nM, respectively (Fig. 5F). However, unexpectedly, we also discovered that I-2 is a much less potent inhibitor against PPZ1_FL_, which includes the ~160-amino-acid N-terminal IDP domain (see Fig. S3 in the supplemental material). With PPZ1_FL_, the IC_50_ of I-2 increases 39-fold to 278 nM; this increase was observed for both recombinant I-2 and I-2 partially purified from rabbit muscle (not shown). (The latter result is consistent with previous studies [29] that showed that *S. cerevisiae* PPZ1_FL_ is also poorly inhibited by I-2.) Furthermore, the specific activities of the two PPZ1 constructs also differ significantly, with 4.6 mU/mg for PPZ1_FL_ and 1,200 mU/mg for PPZ1_cat_. These data suggest that the N-terminal domain likely masks one or more binding sites for I-2 (but not the active site, as the PPZ1_FL_ protein is fully susceptible to MC inhibition [Fig. 3E]) and prevents, via an as yet undetermined mechanism, PPZ1 inhibition by this protein inhibitor.

## DISCUSSION

**FIG 6:**
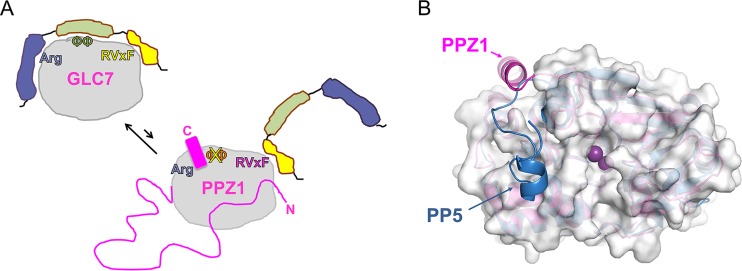
Features of PPZ1 that limit its interaction with GLC7 regulators. (A) Cartoon illustrating that the presence of the disordered N-terminal domain, the lack of conservation of key PP1/GLC7 interaction domains (such as the ΦΦ binding pocket), and the presence of the PPZ1-specific helix negatively impact the binding of PPZ1 with many GLC7-specific regulators, leaving the regulators to preferentially bind and regulate the activity of GLC7. (B) Overlay of PPZ1 (pink) and PP5 (blue; PDB no. 1S95 [30]), illustrating that the C-terminal helices of the two proteins bind different pockets in the core PSP catalytic domain.

Our structural and biochemical studies reveal why, in spite of their high sequence similarity, fungus-specific phosphatases like PPZ1 bind only a subset of the GLC7-specific regulators (Fig. 6A). Specifically, we discovered that there are three distinct mechanisms by which PPZ1 inhibits the binding of many GLC7-specific regulators. First, sequence differences between PPZ1_cat_ and PP1α in canonical SLIM interaction pockets, especially the ΦΦ binding pocket, inhibit the subset of regulators that requires these sites for binding (see Fig. S1 in the supplemental material). Second, the presence of the N-terminal IDP domain, which is not found in GLC7, also negatively impacts the binding of at least some regulators (e.g., I-2), likely through an as yet undefined steric mechanism (Fig. 5 and 6A). Third, structural differences between PPZ1_cat_ and PP1α can also negatively impact regulator binding. In particular, our data show that the presence of the Z1-specific helix hinders access to the C-terminal groove by regulators that bind the Arg site via the ΦΦ site (Fig. 5). In addition, the dynamic nature of this helix raises the intriguing possibility that it may also serve a regulatory role in PPZ1 function. Finally, the binding pocket of the Z1-specific helix is also unique, as that of PP5, the only other known serine/threonine protein phosphatase with a C-terminal helix, binds at the front, versus the top, of the core catalytic domain (Fig. 6B) (30). Together, these results show that instead of PPZ1 competing with GLC7 for its regulators, the regulators preferentially bind and control the activity of GLC7 (Fig. 6A). This resolves a long-standing question about why the presence of the fungus-specific phosphatases does not globally disrupt GLC7 function (12).

Our studies also revealed that multiple pockets in PPZ1_cat_ are unique to this phosphatase and thus can be exploited for drug development. While the PPZ1_cat_ and PP1α active sites are perfectly conserved, the conformation of loop L1 is unique in PPZ1_cat_, as is its folded Z1-specific helix. Both of these new structural elements create novel binding surfaces that are not present in human isoforms of PP1. Thus, they are useful for the development of specific, potent antifungals. Because their active sites are perfectly conserved, one strategy for developing a fungus-specific inhibitor of PPZ1 would be to use “fragment linking” (31): i.e., linking a small molecule that targets one of the unique PPZ1-specific binding pockets with a small molecule that, for example, targets the PP1 active site. This would result in an inhibitor that selectively targets PPZ1, as it would bind PPZ1 with higher affinity than human PP1 isoforms. Because PPZ1 is critical for *C. albicans* hypha formation, a morphological change associated with infectivity, this type of PPZ1-specific inhibitor would stop *C. albicans* infections without killing the commensal pathogen. This would prevent uncontrolled bacterial proliferation that can accompany *C. albicans* elimination and thus will result in novel, potent drugs with minimal side effects for the treatment of candidemia.

## MATERIALS AND METHODS

### Protein expression, purification for pNPP-based assays, and crystallography.

The gene encoding PPZ1_cat_ (aa 171 to 484) was synthesized (GeneArt; Invitrogen), subcloned into the RP1B bacterial expression plasmid (32), and expressed largely as previously described (8). Specifically, the plasmid was cotransformed with the pGRO7 plasmid, which encodes the GroEL/GroES chaperone (TaKaRa), into *E. coli* BL21(DE3) cells (Invitrogen). Cells were grown in LB medium supplemented with 1 mM MnCl_2_ at 30°C to an optical density at 600 nm (OD_600_) of ~0.5, at which point arabinose was added (2 g/liter) to induce the expression of the GroEL/GroES chaperone. At an OD_600_ of ~1, the temperature was lowered to 10°C, and the expression of PPZ1 was induced using 0.1 mM IPTG (isopropyl-β-d-thiogalactopyranoside): the protein was allowed to express for ~20 h at 10°C. The cells were harvested by centrifugation, suspended in fresh LB medium (again supplemented with 1 mM MnCl_2_ and 200 µg/ml of chloramphenicol to inhibit the ribosome), and agitated for ~5 h at 10°C. Harvested cells were frozen and stored at −80°C.

All purifications were performed at 4°C. Cells expressing His_6_-tagged tobacco etch virus (TEV) protease sequence-PPZ1_cat_ were lysed in lysis buffer (25 mM Tris [pH 8.0], 700 mM NaCl, 5 mM imidazole, 1 mM MnCl_2_, 0.1% Triton X-100) using high-pressure homogenization (Avestin C3 EmulsiFlex) in the presence of an EDTA-free protease inhibitor cocktail (Roche). The lysate was clarified by centrifugation at 45,500 × *g*, filtered through a 0.22-µm-pore polyethersulfone (PES) membrane filter (Millipore), and then loaded onto Ni^2+^-nitrilotriacetic acid (NTA) resin (GE Healthcare) preequilibrated in buffer A (25 mM Tris [pH 8.0], 700 mM NaCl, 5 mM imidazole, 1 mM MnCl_2_). Bound His_6_-TEV-PPZ1_cat_ was washed with 100 ml of buffer A, followed by more stringent wash with 100 ml “stringent wash buffer” consisting of 94% buffer A and 6% buffer B (25 mM Tris [pH 8.0], 700 mM NaCl, 250 mM imidazole, 1 mM MnCl_2_). The bound His_6_-TEV-PPZ1_cat_ was eluted using 100% buffer B and immediately purified by size exclusion chromatography (SEC) using Superdex 75 26/60 preequilibrated in 20 mM Tris (pH 8.0), 500 mM NaCl, 0.5 mM Tris(2-carboxyethyl)phosphine (TCEP), and 1 mM MnCl_2_. Fractions containing the His_6_-TEV-PPZ1_cat_ protein were pooled and incubated overnight with TEV protease at 4°C. A second Ni^2+^-NTA “subtraction” purification was used to separate cleaved PPZ1_cat_ from the cleaved His_6_ tag and TEV; the flowthrough, which contained the cleaved PPZ1_cat_, was pooled and purified in a final step using SEC. PPZ1_cat_ was pooled, concentrated, and used immediately for crystallization experiments.

### Protein expression and/or purification for dephosphorylation assays.

The catalytic subunit of rabbit skeletal muscle protein phosphatase 1 (PP1c) was isolated as described (33). The 20-kDa myosin light chain (MLC20) and the myosin light chain kinase (MLCK) were obtained from turkey gizzard, and MLC20 was phosphorylated by MLCK in the presence of [γ-^32^P]ATP and Mg^2+^ as described previously (34). Recombinant 6×His-tagged inhibitor-2 (I-2) was prepared as described previously (28); I-2 was also isolated from rabbit skeletal muscle (35) for some control experiments.

The coding sequences for full-length *Candida* PPZ1_FL_ and its conserved C-terminal catalytic domain, termed PPZ1_cat_, were generated by PCR from the pET28-CaPPZ1 plasmid (GenBank accession no. GQ357913 [14, 36]) and cloned into the *E. coli* expression vector pGEX6p-1 (Amersham Biosciences); both constructs were sequence verified (UD Genomed, Ltd.). Both constructs were then transformed into *E. coli* BL21(DE3) RIL cells (Agilent), and expression of N-terminal glutathione *S*-transferase (GST)-tagged proteins was induced with 0.6 mM IPTG (Sigma) at 18°C. Overnight incubation was used to express GST-PPZ1_cat_, while a shorter, 3-h incubation period was required to reduce inclusion body formation and ensure optimal production of soluble GST-PPZ1_FL_. In both cases, 0.5 mM MnCl_2_ was included in the culture medium, and 1 mM MnCl_2_ was present during the subsequent purification steps. The fusion proteins were purified using glutathione-Sepharose 4B (GE Healthcare) resin, and the GST tag was removed using the PreScission protease (GE Healthcare) during elution (following the instructions of the manufacturer). The protein concentration was determined using the Bradford assay, and protein purity was verified by SDS-PAGE.

### Protein phosphatase assays.

The activities of the recombinant *C. albicans* phosphatases were measured with ^32^P-labeled MLC20 substrate as previously described (34), with the exception that the substrate concentration was 1 µM and 2 mM MnCl_2_ was included in the assay mixtures. The activity of rabbit PP1 was also determined under identical conditions as a control. The 50% inhibitory concentrations (IC_50_s) of microcystin-LR (obtained from Enzo Life Sciences or purified as previously described [37]) were determined by using either the protein substrate or a small molecule substrate as described previously (38), except that pNPP (Sigma-Aldrich) was used instead of 3-*O*-methylfluorescein phosphate (OMFP). Reactions were carried out in 96-well plates (Costar). MC concentrations were prepared by serial dilution and added to 150 µl of buffered enzyme (18 nM protein in 40 mM HEPES [pH 7.0], 1.33 mM dithiothreitol [DTT], 1.33% [vol/vol] Triton X-100, 0.133 mg/ml bovine serum albumin [BSA], 1.33 mM sodium ascorbate, 1.33 mM MnCl_2_) in wells containing the reaction mixtures. The high-signal controls were prepared by adding dimethyl sulfoxide (DMSO), matching the amount present in the reaction mixture with the highest concentration of MC. The low-signal controls were the reaction mixtures containing the highest concentration of microcystin-LR. The reactions were initiated by the addition of pNPP substrate and incubated at room temperature for 30 min. The reactions were stopped using 100 µl of 300 mM potassium phosphate (pH 10). The absorbance was measured at 405 nm using an Epoch spectrophotometer (BioTek). The percentage of activity of each protein was calculated using the equation [(absorbance − low-signal control)/(high-signal control − low signal control)] × 100%. The IC_50_ was then calculated using SigmaPlot 12.5.

### Crystallization and structure determination.

Crystals of apo-PPZ1_cat_ were obtained using hanging drop vapor diffusion in 1.8 M ammonium citrate tribasic (pH 7.0) in a 2:1 protein/crystallization condition ratio. To generate PPZ1_cat_-MC crystals, PPZ1_cat_ was first incubated with MC at a 1:1 molar ratio for 15 min before crystallization using hanging drop vapor diffusion in 0.06 M citric acid (pH 4.1) plus 16% (wt/vol) polyethylene glycol (PEG) 3350. For data collection, crystals were first cryo-protected with 30% glycerol in mother liquor (PPZ1_cat_) or 3.4 M Na-malonate (pH 4.0) (PPZ1_cat_-MC) and then flash-frozen in liquid nitrogen. X-ray data were collected in house at 100 K using a Rigaku FR-E+ Superbright rotating copper anode X-ray generator with a Saturn 944+ HG charge-coupled device (CCD) detector (Brown University Structural Biology Facility). The data were phased using molecular replacement (Phaser as implemented in PHENIX [39]) using PP1 (PDB no. 4MOV [19]) as the search model. Clear electron density of MC could be observed bound to the active site. The initial models were built using Phenix.AutoBuild (40) followed by iterative rounds of refinement in PHENIX and manual building using Coot (41).

### Isothermal titration calorimetry.

GADD34_552–567_ peptide was purchased from Biosynthesis, Lewisville, TX. The peptide was dissolved directly in ITC buffer (20 mM Tris [pH 8.0], 500 mM NaCl, 0.5 mM TCEP, 1 mM MnCl_2_). GADD34_552–567_ was titrated into PPZ1_cat_ using a VP-ITC microcalorimeter at 25°C (Microcal, Inc.). Data from the ITC runs were analyzed using Origin 7.0.

### Pulldown assay.

GADD34_552–567_ was purchased. PNUTS_394–433_ and spinophilin_417–602_ were purified according to a method described previously (19, 27). Spinophilin_417–602_ ΦΦ→AA, R469D, and R469E mutants were generated using site-directed mutagenesis. PP1α_7–330_ and PPZ1_cat_ were purified by the method described above but retained the His tag. After SEC (20 mM Tris [pH 8.0], 500 mM NaCl, 0.5 mM TCEP, 1 mM MnCl_2_), 50% (vol/vol) glycerol was added to both PP1α_7–330_ and PPZ1_cat_. The final concentrations of the proteins were adjusted to 5 µM. Ten micromoles of each PP1 regulator was added into 500 µl of PP1α_7–330_ or PPZ1_cat_. The mixture was incubated under rocking (4°C) for 60 min to allow for complex formation. A 15-μl bed volume of Ni-NTA resin (GE Healthcare) was added into tubes, and the mixture was incubated at 4°C for 60 min. The beads were pelleted by centrifugation (2000 × *g*) and washed three times with 500 µl of SEC buffer. Forty microliters of SDS loading buffer was added to the beads, and the samples were boiled at 80°C for 5 min and analyzed on NuPAGE 4 to 12% bis-Tris gels. Gels were stained overnight with SYPRO ruby protein gel stain (Life Technologies) according to the manufacturer’s protocols and scanned using a Typhoon 9410 laser scanner (GE Healthcare) with an excitation wavelength of 457 nm and emission filter of 610 nm following destaining. Densitometry was performed using ImageQuant TL 7.0 software for quantification of the band intensity.

### Accession number(s).

Atomic coordinates and structure factors have been deposited in the Protein Data Bank under accession no. 5JPE for PPZ1 and 5JPF for PPZ1-MC.

## SUPPLEMENTAL MATERIAL

Figure S1  Conservation of PP1 interaction pockets between *Hs*PP1α (1α) and *Ca*PPZ1 (Z1). The residues that define the metal, toxin, and regulatory motif binding pockets in *Hs*PP1α and the corresponding residues in CaPPZ1 are shown. Residues that are different between the two proteins are in boldface and shaded in a darker color. The sequence identity (ID) and similarity (Sim) of the distinct binding pockets are calculated above each list. Download Figure S1, TIF file, 2.2 MB

Figure S2  MC potently inhibits recombinant *Hs*PP1α and *Ca*PPZ1 activity measured with pNPP. The data represent the mean ± standard deviation (SD) from 4 experiments. Download Figure S2, TIF file, 0.1 MB

Figure S3  Secondary structure prediction for *Ca*PPZ1. The N-terminal domain (gray box) is predicted to lack secondary structural elements (pink cylinders, α-helices; yellow arrows, β-strands), while the catalytic domain (pink box) is predicted to be structured. The C-terminal residues in PPZ1 are predicted to be helical (green box). “Conf” represents the confidence of the prediction, with higher bars indicating greater confidence scores. Download Figure S3, TIF file, 1.8 MB

Figure S4  Mutation of the spinophilin ΦΦ and Arg motifs negatively impacts its ability to bind PP1α. Pull-down assays with (A) PP1α or (B) PPZ1 and either wild-type (wt) spinophilin or spinophilin variants in which the ΦΦ or Arg PP1 interaction motifs are mutated (Spino_ΦΦ→AA_, Spino_R469D_, and Spino_R469E_). (C) Densitometry and quantification of pulldown results shown in panels A and B. PP1α is in blue and PPZ1 in red. Mutation of either the ΦΦ or Arg motif in spinophilin reduces the amount of spinophilin pulled down by PP1α to levels nearly identical to those obtained with PPZ1. Download Figure S4, TIF file, 1.1 MB
