# Supplementary figures and images for: Molecular Insights into the Fungus-Specific Serine/Threonine Protein Phosphatase Z1 in Candida albicans
Source: mBio. 2016 Aug 30;7(4):e00872-16. doi: 10.1128/mBio.00872-16 (PMC4999541; doi:10.1128/mBio.00872-16)

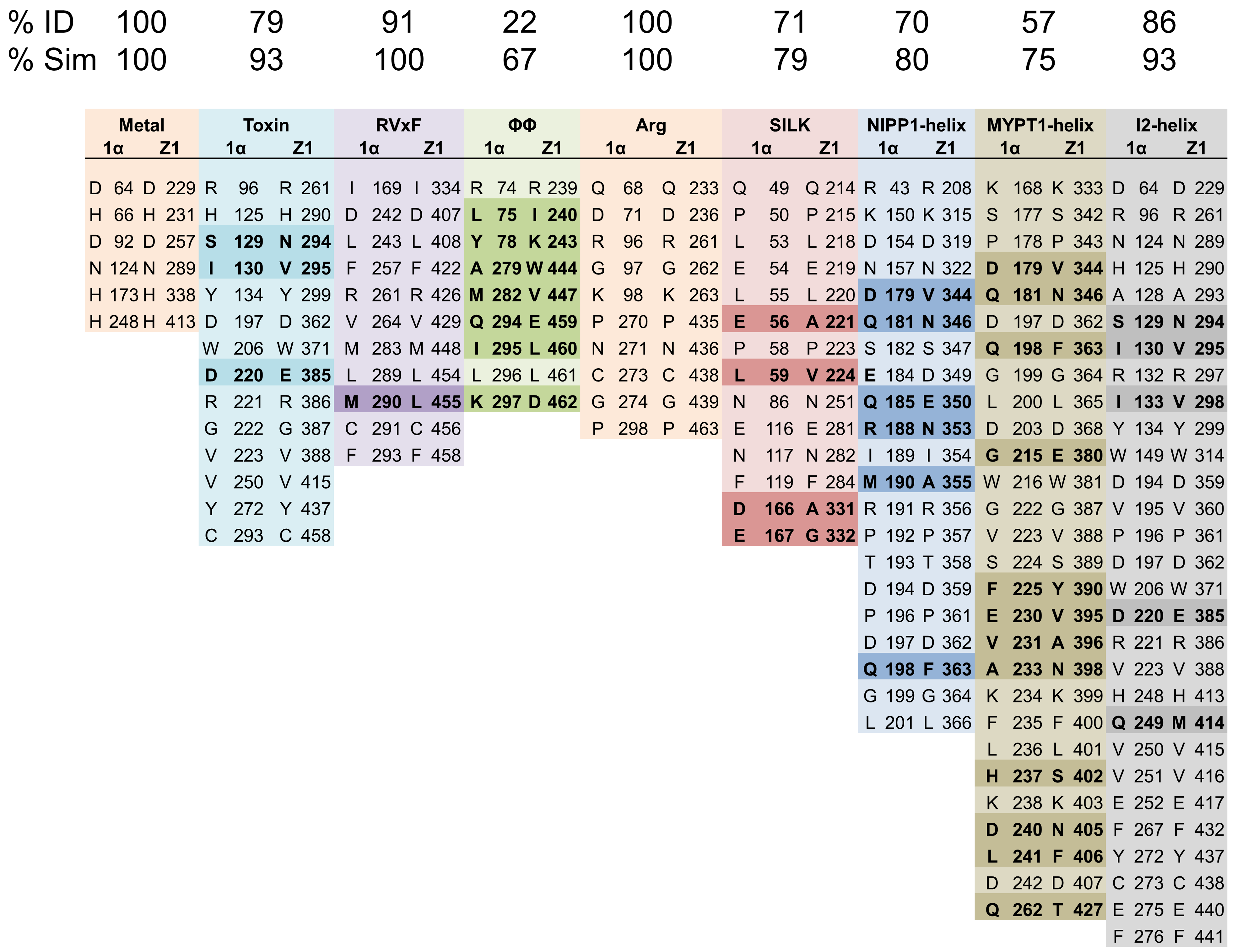

Supplement: Figure S1 — Conservation of PP1 interaction pockets between HsPP1α (1α) and CaPPZ1 (Z1). The residues that define the metal, toxin, and regulatory motif binding pockets in HsPP1α and the corresponding residues in CaPPZ1 are shown. Residues that are different between the two proteins are in boldface and shaded in a darker color. The sequence identity (ID) and similarity (Sim) of the distinct binding pockets are calculated above each list. Download [file mbo004162959sf1.tif]

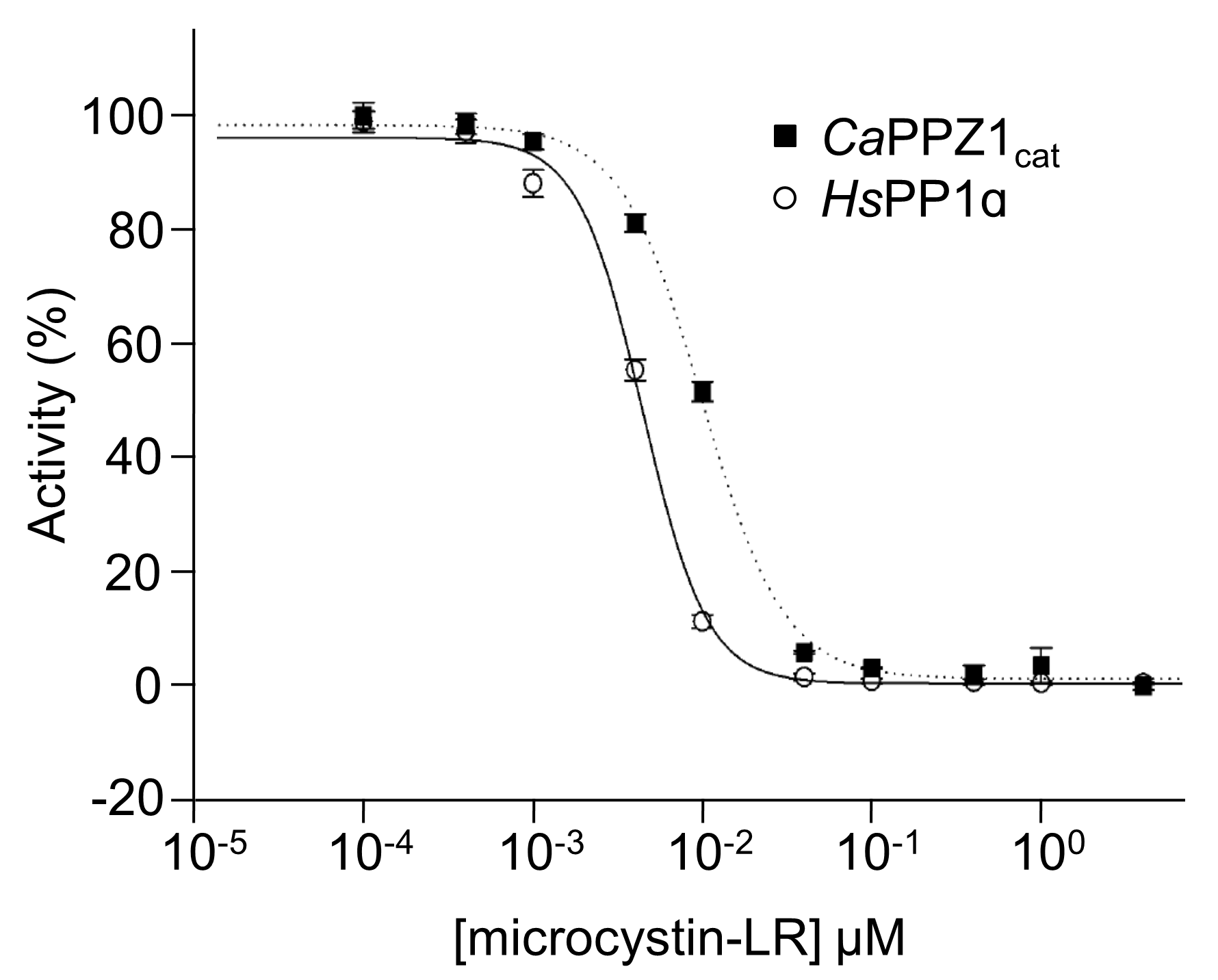

Supplement: Figure S2 — MC potently inhibits recombinant HsPP1α and CaPPZ1 activity measured with pNPP. The data represent the mean ± standard deviation (SD) from 4 experiments. Download [file mbo004162959sf2.tif]

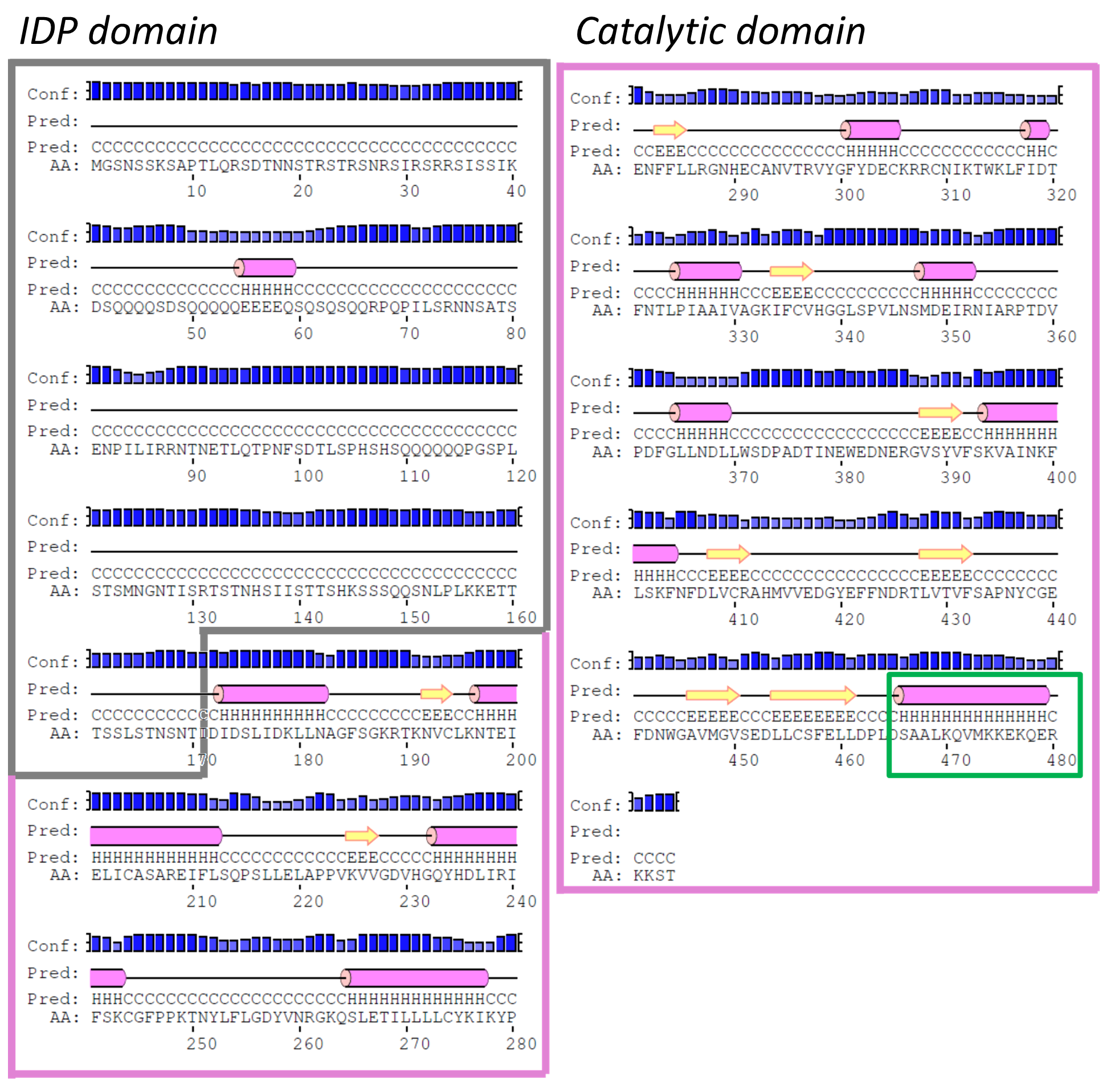

Supplement: Figure S3 — Secondary structure prediction for CaPPZ1. The N-terminal domain (gray box) is predicted to lack secondary structural elements (pink cylinders, α-helices; yellow arrows, β-strands), while the catalytic domain (pink box) is predicted to be structured. The C-terminal residues in PPZ1 are predicted to be helical (green box). “Conf” represents the confidence of the prediction, with higher bars indicating greater confidence scores. Download [file mbo004162959sf3.tif]

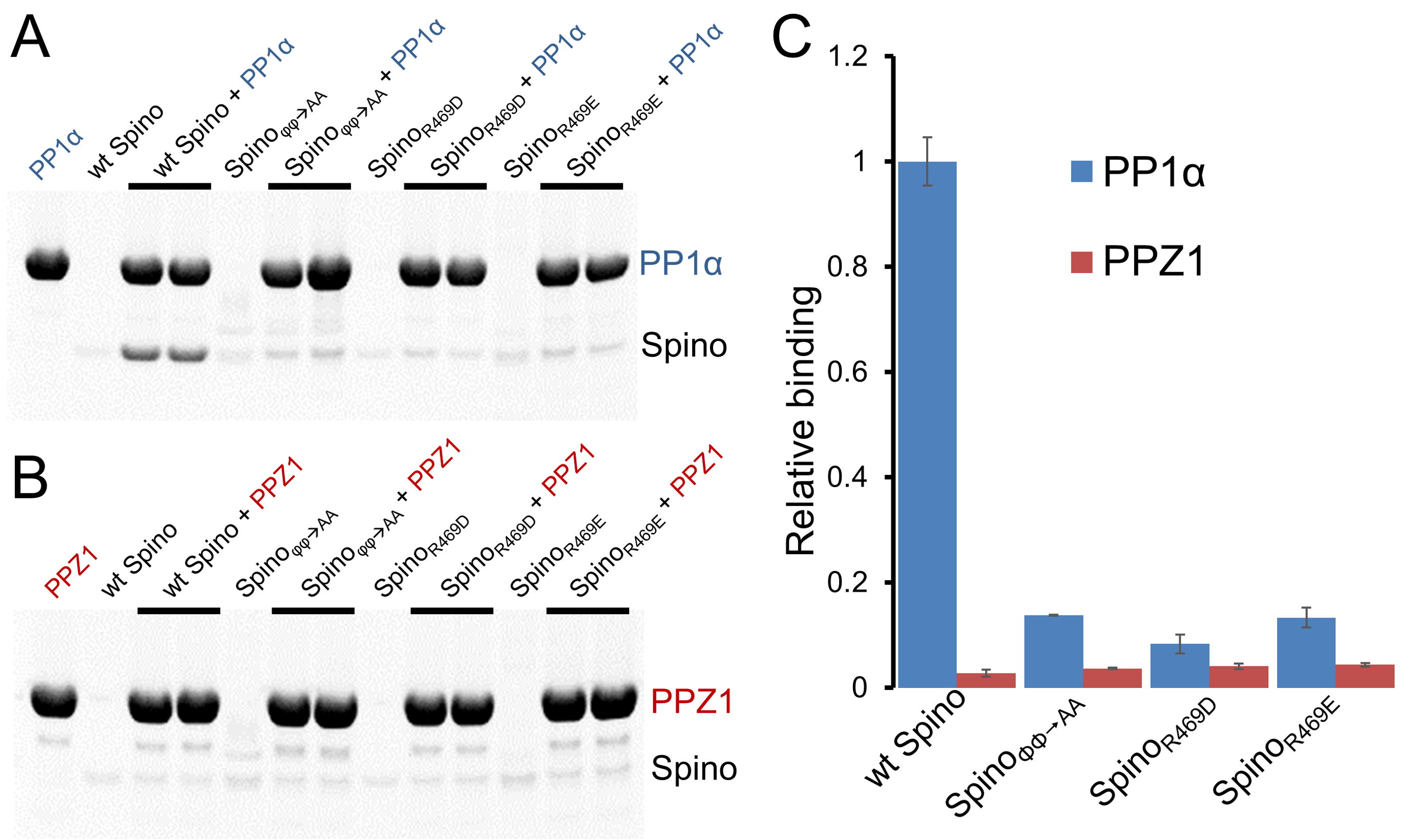

Supplement: Figure S4 — Mutation of the spinophilin ΦΦ and Arg motifs negatively impacts its ability to bind PP1α. Pull-down assays with (A) PP1α or (B) PPZ1 and either wild-type (wt) spinophilin or spinophilin variants in which the ΦΦ or Arg PP1 interaction motifs are mutated (SpinoΦΦ→AA, SpinoR469D, and SpinoR469E). (C) Densitometry and quantification of pulldown results shown in panels A and B. PP1α is in blue and PPZ1 in red. Mutation of either the ΦΦ or Arg motif in spinophilin reduces the amount of spinophilin pulled down by PP1α to levels nearly identical to those obtained with PPZ1. Download [file mbo004162959sf4.tif]
